# Comparison of the Diagnostic Performance of Steatosis Indices for Discrimination of CT-Diagnosed Metabolic Dysfunction-Associated Fatty Liver Disease

**DOI:** 10.3390/metabo12070664

**Published:** 2022-07-19

**Authors:** A. Lum Han, Hee Kyung Lee

**Affiliations:** Department of Family Medicine, Wonkwang University Hospital, 895 Muwang-ro, Iksan 54538, Korea; yebbnkim@gmail.com

**Keywords:** metabolic dysfunction-associated fatty liver disease, liver, non-alcoholic fatty liver disease, non-alcoholic fatty liver disease diagnostic imaging

## Abstract

Non-alcoholic fatty liver disease (NAFLD) was redefined as metabolic dysfunction-associated fatty liver disease (MAFLD) in 2020. Due to this, further validation of the non-invasive tests used in NAFLD diagnosis is required for MAFLD. There are five known steatosis indices for computed tomography (CT)-diagnosed MAFLD. These indices include the fatty liver index (FLI), the hepatic steatosis index (HSI), the lipid accumulation product (LAP), the visceral adiposity index (VAI), and the Zhejiang University index (ZJU). We aimed to analyze the diagnostic abilities of these five widely known steatosis indices for CT-diagnosed MAFLD. From March 2012 to October 2019, we retrospectively analyzed the clinical information and images of 1300 adults aged ≥19 years who underwent CT scans at our institution. To compare differences, the Chi-square test and independent *t*-test were used for categorical and continuous variables, respectively. The area under the receiver operating characteristic (AUROC) curve was used to validate the diagnostic accuracy of MAFLD. Of the five indices, FLI was the best at predicting MAFLD, with the highest AUROC (0.791). The sensitivity and specificity of FLI for diagnosing MAFLD were both 70.9%. The optimal cut-off value was 29.9. FLI is a useful surrogate index for screening MAFLD in clinical practice.

## 1. Introduction

Non-alcoholic fatty liver disease (NAFLD) is the most common chronic liver disease worldwide. The prevalence of NAFLD is gradually increasing with the rising incidence of obesity and diabetes, which are both associated with westernized lifestyle changes. This is causing social and economic issues in the global healthcare system [1]. NAFLD is considered the hepatic component of metabolic syndrome and increases liver-related mortality. NAFLD can develop into hepatocellular carcinoma without liver cirrhosis and is expected to become the leading cause of liver transplantation. NAFLD is a multisystemic disease, and extrahepatic complications, such as cardiovascular diseases and various cancers, comprise its major complications. Therefore, early detection of NAFLD is crucial for preventing its progression to advanced diseases [2,3,4,5].

The Asian Pacific Association for the Study of the Liver (APASL) redefined NAFLD as metabolic dysfunction-associated fatty liver disease (MAFLD) in 2020. MAFLD is diagnosed if there is overweight/obesity, diabetes mellitus (DM), or metabolic risk abnormalities, along with fatty liver diagnosed by histological biopsy, imaging, or blood scores. Unlike NAFLD, which is diagnosed via exclusion, MAFLD applies positive diagnostic criteria that do not exclude alcohol intake or other liver diseases [6].

Following this redefinition, further validation of the non-invasive tests used in NAFLD diagnosis is required for MAFLD. Biopsy-free scoring systems, including the indices widely used in fatty liver evaluation and NAFLD diagnosis, should be validated. The Zhejiang University index (ZJU) was developed in China in 2015 and consists of body mass index (BMI), fasting plasma glucose (FPG), triglyceride (TG), and the alanine aminotransferase (ALT) to aspartate aminotransferase (AST) (ALT/AST) ratio [7]. The visceral adiposity index (VAI) was developed in Italy in 2010 and consists of waist circumference (WC), body mass index (BMI), TG, and high-density lipoprotein cholesterol (HDL-C). It indirectly indicates visceral adipose function and is strongly related to cardiometabolic risk [8]. The hepatic steatosis index (HSI) was developed in South Korea in 2010 and is based on ultrasonography (US), and its components are the ALT/AST ratio, BMI, and DM [9]. The lipid accumulation product (LAP) was introduced in 2005 as an index using the National Health and Nutrition Examination Survey (NHANES) III data and consists of WC and TG. It has been reported that it predicts cardiovascular disease risk better than BMI [10]. The fatty liver index (FLI) is an algorithm introduced in 2006 by Bedogni et al. in Italy. It is calculated using BMI, WC, TG, and gamma-glutamyl transferase (GGT) data. It was initially developed for US and intended for NAFLD risk stratification. However, it has been verified as a diagnostic tool and is generally used in large-scale epidemiological studies [11,12]. The components of steatosis indices are summarized (Table 1).

A biopsy is the gold standard for diagnosing fatty liver disease. However, it may be invasive and result in complications such as bleeding [13]. Abdominal US is the most used imaging test for diagnosing fatty liver disease in clinical practice. However, it is less reproducible and examiner-dependent. Moreover, the evaluation of obese patients is limited, and mild steatosis is challenging to detect. Magnetic resonance imaging (MRI) was not easily accessible and was used for research purposes in the past, but it is now the most definitive imaging modality for qualitative and quantitative evaluation of fatty liver. Computed tomography (CT) is more specific than abdominal US in diagnosing steatosis but is more expensive and involves radiation exposure. Thus, it is often used when another deep organ evaluation is required [2,14,15].

To the best of our knowledge, there have been no studies on MAFLD using CT and hepatic steatosis indices. We aimed to select the CT-diagnosed MAFLD group; therefore, we conducted a comparative analysis of five widely studied indices’ diagnostic capabilities, namely, FLI, HSI, LAP, VAI, and ZJU.

## 2. Results

### 2.1. Participants

Table 2 presents the general characteristics of the 1300 included participants. The mean age was approximately 52 years in both non-MAFLD and MAFLD groups. Of the 1300 participants, 447 (337 men, 110 women; ≈3:1) were in the MAFLD group. BMI, blood pressure, FPG, AST, ALT, GGT, TG, Cr, and UA levels were significantly higher, whereas HDL-C was significantly lower in the MAFLD group compared with the non-MAFLD group. GGT levels were 37 ± 66 (non-MAFLD group) versus 67 ± 176 (MAFLD group) (*p* = 0.001). Of the lipid profiles, only the differences in TG and HDL-C levels were significant (*p* < 0.001); TG and HDL-C levels were 99 ± 65 and 57 ± 13, respectively, in the non-MAFLD group versus 155 ± 143 and 51 ± 13 in the MAFLD group. Serum UA levels were 5.0 ± 1.4 in the non-MAFLD group versus 5.8 ± 1.4 in the MAFLD group (*p* < 0.0001). ZJU, VAI, HSI, LAP, and FLI were significantly higher in the MAFLD group (*p* < 0.0001) than in the non-MAFLD group.

### 2.2. AUROC Analysis for MAFLD

**Figure 1:**
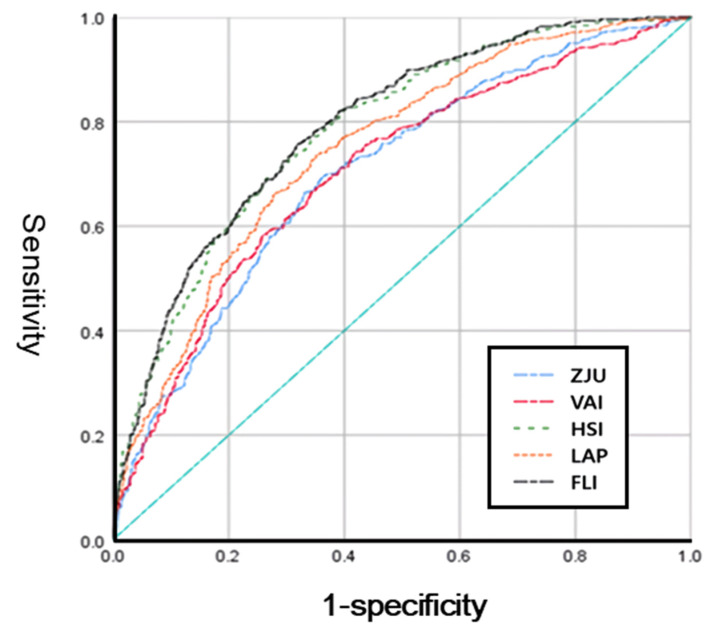
Receiver operating characteristic curves for ZJU (blue), VAI (pink), LAP (orange), HSI (green), and FLI (black) for identifying MAFLD. The diagonal line is the reference line. MAFLD, metabolic dysfunction-associated fatty liver disease; ZJU, Zhejiang University index; VAI, visceral adiposity index; HSI, hepatic steatosis index; LAP, lipid accumulation product; FLI, fatty liver index.

In the AUROC analysis (Figure 1, all five indices showed an acceptable performance index of >0.7. Of these indices, FLI showed the best performance, with an AUROC of 0.791 (95% confidence interval (CI): 0.766–0.816). The optimal cut-off value was 29.9. The sensitivity and specificity were both 70.9%. The AUROC of HSI was 0.784 (95% CI: 0.758–0.809), and the sensitivity, specificity, and cut-off values were 71.5%, 70.9%, and 33.2, respectively. The AUROC values decreased in the order of 0.747, 0.705, and 0.704 for LAP, VAI, and ZJU, respectively (Table 3).

### 2.3. Diagnostic Accuracy for MAFLD by Sex

Table 4 shows the results of the subgroup analysis according to sex. The AUROC of HSI was 0.773 (95% CI: 0.740–0.806) in men and 0.793 (95% CI: 0.749–0.838) in women. Women showed better results than men. Of the five indices, only HSI showed more accurate results for women than men, and its value was the highest in the sex analysis results. In comparison, FLI showed a lower AUROC value than HSI; however, there was little difference between sexes.

### 2.4. Diagnostic Accuracy for MAFLD by Age

The performance of each index was assessed according to age group. The AUROC values of HSI were 0.862 and 0.748 for those aged ≤40 years and >60 years, respectively. HSI showed better AUROC values, followed by FLI. In contrast, FLI showed better results than HSI in the population aged 41–60 years. In the age-specific analysis, FLI showed the highest AUROC value of 0.849 in the population aged 41–50 years, while HSI showed the highest AUROC value of 0.862 in those aged ≤40 years. For all five indices, AUROC values tended to decrease in those aged >50 years compared with those in the other age groups. In particular, LAP, VAI, and ZJU were lower in the population aged >60 years than in the other age groups, with predictive values of 0.628–0.667 (Table 5).

## 3. Discussion

This study is the first to compare the diagnostic performance of relatively well-known hepatic steatosis indices for CT-diagnosed MAFLD. All five indices showed adequate performance in general; however, FLI was the best, with the highest AUROC value.

To date, only a few studies have investigated the association between MAFLD and steatosis indices. In a recent study using US as a reference standard and NHANES III, FLI (AUROC, 0.793) was the best, demonstrating values similar to those in this study. This was followed by HSI (0.764) and VAI (0.724). The BMI categories were normal (BMI < 25), overweight (25 to <30), and obesity (BMI ≥ 30) [16].

According to a large-scale MAFLD study using US as a reference method in China, the AUROC of FLI for predicting hepatic steatosis was 0.856 (95% CI: 0.854–0.859) in men and 0.909 (95% CI: 0.906–0.911) in women, showing excellent performance. In women, FLI exhibited outstanding predictive abilities. The optimal cut-off values for hepatic steatosis were 37.25 and 17.00 for men and women, respectively, following sex analysis. In the AUROC analysis of MAFLD, FLI values were 0.870 and 0.923 for men and women, respectively, showing improved diagnostic ability compared to hepatic steatosis. The authors demonstrated that FLI could predict MAFLD better than hepatic steatosis because it reflects WC, BMI, and TG, which are components of the MAFLD diagnostic criteria. MAFLD also showed good predictive ability in women. The BMI criteria were applied in the same manner as in our previous study [17].

Next, we examined the relationship between existing NAFLD and the indices before MAFLD. A review article stated that FLI can be used to screen NAFLD in high-risk populations and is useful for detecting the risk of atherosclerosis, incidental diabetes, and chronic kidney disease [18]. In our previous study on CT-diagnosed NAFLD and FLI, the AUROC of FLI was 0.696. Compared to the results of this study, it can be observed that FLI improves MAFLD [19]. Subjects with MAFLD present with a higher BMI and WC and a worse metabolic state than those with NAFLD [20]. Compared to the role of FLI in the diagnosis of NAFLD, FLI is more compatible with MAFLD. One study reported that GGT, a component of FLI, may play a role in discriminating between NAFLD and MAFLD [21]. In a study conducted using CT in Brazil, both FLI and HSI showed the same AUC (0.76) [22]. A study conducted on severely obese middle-aged North American women, including patients with a BMI ≥35, compared ZJU, HSI, LAP, and VAI based on the criteria of a CT liver/spleen attenuation ratio of <1.1. The AUROC of ZJU was 0.742, the highest value among indices [23].

In a study of the general population in China, 7324 patients with NAFLD were diagnosed using US. The AUROC of ZJU was 0.925, indicating good performance in both men and women. ZJU is an algorithm developed in China, and many validation studies have been conducted on the Chinese population. Hence, it seems to reflect Chinese characteristics well. In addition, in the analysis by age group, ZJU showed a high agreement of 0.954 for those aged >60 years, followed by FLI, with an AUROC value of 0.873. FLI was 0.894 in patients ≤30 years, the highest value among the indices [24]. Another study based on US in Western China showed that the AUROC of FLI was 0.880, followed by ZJU (0.861), LAP (0.853), and HSI (0.833); all indices showed good performance [25]. A study in eastern China reported FLI (0.852), ZJU (0.847), LAP (0.835), and VAI (0.791). In the sex analysis, all indices showed good predictive values for women. For FLI, the total cut-off value was 20.6, and according to sex, it was 25.3 for men and 8.4 for women [26]. According to a Japanese study, ZJU (0.886), FLI (0.884), and HSI (0.874) showed promising results. Analysis by sex revealed that ZJU showed higher AUROC than FLI in men and women, whereas FLI showed better results than ZJU in DM patients [27]. In the Bagnacavallo study in Italy, FLI, LAP, HSI, and ZJU showed similar diagnostic abilities for hepatic steatosis and NAFLD, of which FLI had some advantages. For all indices, AUROC values were somewhat lower in NAFLD than in hepatic steatosis [28]. This contrasts with the higher AUROC in MAFLD than in hepatic steatosis in a previous MAFLD study [17]. In a Korean study, FLI in US-diagnosed NAFLD was found to have an overall cut-off value of 29 and an AUROC of 0.82 [29].

The difference in the accuracy of indices between studies is due to differences in the derived cohorts and the race, ethnicity, sex, and age distributions of the sample populations. In addition, personal factors, including underlying diseases such as diabetes and differences in diagnostic criteria for NAFLD and MAFLD, imaging modalities, and BMI diagnostic criteria, are also thought to have an impact. Thus, to increase diagnostic accuracy, it is necessary to use an algorithm that considers the characteristics of the population. Considering the above results, applying customized models for each country, region, age, and sex is necessary. It seems reasonable to consider a specific cut-off value rather than uniformly applying the established cut-off value.

On the other hand, in terms of the pathophysiology of NAFLD, oxidative stress plays a key role with insulin resistance in liver damage. As a cause of oxidative stress, gut dysbiosis increases intestinal mucosa permeability and enhances the gut–liver axis with bacterial overgrowth and endotoxin [30]. The effects of oxidative stress in MAFLD are still unclear. However, it could also influence the pathophysiology of MAFLD. Further research is needed for MAFLD.

This study had several limitations. First, it was a cross-sectional retrospective study from a single center and did not represent the population. However, we attempted to overcome this limitation by using a sample size of 1300. Second, CT, not biopsy, was used to diagnose fatty liver. Biopsy, a confirmatory test, cannot be readily performed in clinical settings. Although US is mostly used for diagnosing fatty liver in clinical practice, we improved its accuracy by evaluating fatty liver with CT, which can be quantitatively assessed. Third, in this study, the optimal cut-off values for men and women could not be obtained. Further research is required to analyze this.

During the COVID-19 pandemic, the incidence of newly diagnosed MAFLD increased. Bidirectionally, MAFLD is associated with COVID-19 severity [31]. Thus, it is necessary to implement multidisciplinary management, including lifestyle modification, through early screening with awareness of MAFLD, which is a multifactorial disease in the current clinical field. To this end, future studies should focus on the well-designed validation of large-scale samples by utilizing more advanced imaging modalities and non-invasive markers to diagnose MAFLD and identify high-risk patients.

## 4. Materials and Methods

### 4.1. Study Population

**Figure 2:**
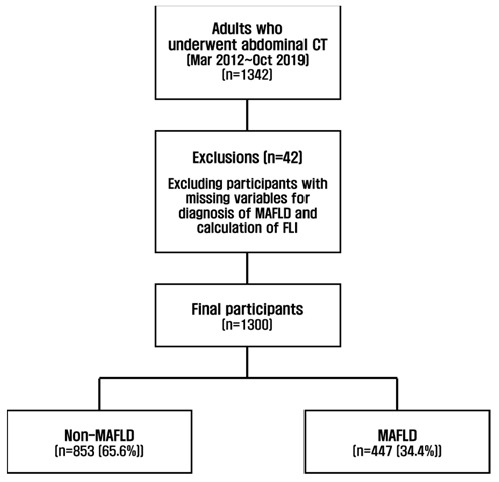
Flowchart of participants’ inclusion and exclusion.

We retrospectively analyzed the clinical information and images of adults aged ≥19 years who underwent abdominopelvic CT scans at our institution. Of the total 1342 patients, 42 with missing variables necessary for MAFLD diagnosis and FLI calculation were excluded. Subsequently, 1300 participants (774 men and 526 women) were included (Figure 2. This study complied with the research ethics guidelines of the Declaration of Helsinki and was approved by the Wonkwang University Hospital Institutional Review Board (approval number 2021-09-007).

### 4.2. Diagnosis of MAFLD

MAFLD was diagnosed based on the presence of hepatic steatosis on CT examination, along with one of the preceding three criteria as per the APASL statement [6].

### 4.3. Diagnosis of Fatty Liver

Abdominal CT scanning was performed using the SOMATOM Definition (Siemens Medical Solutions, Forchheim, Germany). Experienced radiologists blinded to the patient’s medical information performed the CT image interpretation and diagnosed steatosis. Hepatic steatosis was diagnosed when the liver attenuation value was <40 Hounsfield units (HU) or <10 HU when compared with that of the spleen.

### 4.4. Anthropometric and Biochemical Measurements

BMI was calculated by dividing body weight (kg) by height squared (m^2^). Applying the Asian criteria, BMI ≥ 23 was classified as overweight, and BMI ≥ 25 was classified as obese. WC, one of the FLI variables, was measured at the lowest rib and iliac crest midpoint on the CT image. After overnight fasting for more than 8 h, blood specimens were collected from each participant’s veins. They were immediately sent to Neodine Lab and analyzed. FPG, AST, ALT, GGT, total cholesterol, TG, HDL-C, low-density lipoprotein cholesterol (LDL-C), creatinine (Cr), and uric acid (UA) levels were measured.

### 4.5. Calculations of Steatosis Indices: Formulae

Reference [1]

ZJU = BMI (kg/m^2^) + FPG (mmol/L) + TG (mmol/L) + (3 × ALT/AST Ratio) (+2, If Women) (1)

Reference [2]

VAI = [WC/39.68 + (1.88 × BMI)] × (TG/1.03) × (1.31/HDL) for Men; [WC/36.58 + (1.89 × BMI)] × (TG/0.81) × (1.52/HDL) for Women(2)

Reference [3]

HSI = 8 × (ALT/AST Ratio) + BMI (+2, If Women; +2, If DM)(3)

Reference [4]

LAP = (WC − 58) × TG for Women; (WC − 65) × TG for Men(4)

Reference [5]

FLI = (e^0.953 × Loge (TG) + 0.139 × BMI+ 0.718 × Loge (GGT) + 0.053 × WC − 15.745^)/(1 + e^0.953 × Loge (TG) + 0.139 × BMI+ 0.718 × Loge (GGT) + 0.053 × WC − 15.745^) × 100 (5)

### 4.6. Statistical Analyses

The Chi-square test and independent *t*-test were used to compare differences in the general characteristics and the five indices according to the presence of MAFLD. A receiver operating characteristic (ROC) curve was drawn to determine the optimal cut-off point. Sensitivity and specificity were calculated. The area under the ROC curve (AUROC) was used to evaluate the diagnostic accuracy. Statistical significance was set at a *p*-value < 0.05. All statistical analyses were performed using SPSS for Windows version 26.0 (SPSS Inc., Chicago, IL, USA).

## 5. Conclusions

In summary, we investigated the diagnostic performance of FLI, HSI, LAP, VAI, and ZJU as common steatosis indices for identifying MAFLD using CT. The area under the receiver operating characteristic (AUROC) curve was used to validate the diagnostic accuracy of MAFLD. HSI showed good performance for detecting MAFLD in older patients. Of the five indices, FLI was the best at predicting MAFLD, with the highest AUROC (0.791). The sensitivity and specificity of FLI for diagnosing MAFLD were both 70.9%. The optimal cut-off value was 29.9. FLI is a useful surrogate index for screening MAFLD in clinical practice.

## Figures and Tables

**Table 1:** The components of common steatosis indices.

	Biochemical Markers	Other Variables
Zhejiang University index (ZJU)	FPG, TG, ALT/AST ratio	BMI
Visceral adiposity index (VAI)	TG, HDL-C	BMI, WC
Hepatic steatosis index (HSI)	ALT/AST ratio	BMI
Lipid accumulation product (LAP)	TG	WC
Fatty liver index (FLI)	TG, GGT	BMI, WC

FPG, fasting plasma glucose; TG, triglyceride; ALT/AST ratio, alanine aminotransferase to aspartate aminotransferase ratio; HDL-C, high-density lipoprotein cholesterol; GGT, gamma-glutamyl transferase; BMI, body mass index; WC, waist circumference.

**Table 2:** General characteristics of the study population.

Total Participants (n = 1300)		MAFLD		*p*-Value
		No (n = 853)	Yes (n = 447)	
Sex	Male	437 (51.2)	337 (75.4)	<0.0001
	Female	416 (48.8)	110 (24.6)	
Age (years)		52 ± 11	52.1 ± 9.7	0.0429
BMI (kg/m^2^)		23.5 ± 2.8	26.6 ± 3.2	<0.0001
SBP (mmHg)		122 ± 13	127 ± 12	<0.0001
DBP (mmHg)		75.5 ± 9.6	79.9 ± 9.8	<0.0001
FPG (mg/dL)		98 ± 48	106 ± 30	0.004
AST (IU/L)		30 ± 19	38 ± 40	<0.0001
ALT (IU/L)		25 ± 21	41 ± 46	<0.0001
GGT (IU/L)		37 ± 66	67 ± 176	0.001
TC (mg/dL)		202 ± 76	204 ± 44	0.668
TG (mg/dL)		99 ± 65	155 ± 143	<0.0001
HDL-C (mg/dL)		57 ± 13	51 ± 13	<0.0001
LDL-C (mg/dL)		121 ± 60	123 ± 37	0.517
Creatinine (mg/dL)		0.79 ± 0.20	0.85 ± 0.17	<0.0001
Uric Acid (mg/dL)		5.0 ± 1.4	5.8 ± 1.4	<0.0001
ZJU		224 ± 86	291 ± 150	<0.0001
VAI		138 ± 122	251 ± 302	<0.0001
HSI		31.4 ± 4.0	36.0 ± 4.8	<0.0001
LAP		2049 ± 1883	4352 ± 6312	<0.0001
FLI		23 ± 21	49 ± 26	<0.0001

MAFLD, metabolic dysfunction-associated fatty liver disease; SBP, systolic blood pressure; DBP, diastolic blood pressure; BMI, body mass index; FPG, fasting plasma glucose; GGT, gamma-glutamyl transferase; TC, total cholesterol; HDL-C, high-density lipoprotein cholesterol; TG, triglyceride; ALT, alanine aminotransferase; AST, aspartate aminotransferase; LDL-C, low-density lipoprotein cholesterol; ZJU, Zhejiang University index; VAI, visceral adiposity index; HSI, hepatic steatosis index; LAP, lipid accumulation product; FLI, fatty liver index. Data are expressed as number (percentage) or mean ± standard deviation. *p*-values were determined using the Chi-square test and the independent *t*-test.

**Table 3:** Diagnostic performances of ZJU, VAI, HSI, LAP, and FLI for detecting MAFLD.

	AUROC (95% CI)	*p*-Value	Sensitivity (%)	Specificity (%)	Cut-Off Value
ZJU	0.704 (0.675–0.734)	<0.0001	66.4	66.5	231.1261
VAI	0.705 (0.675–0.735)	<0.0001	66.0	65.8	142.9275
HSI	0.784 (0.758–0.809)	<0.0001	71.5	70.9	33.2182
LAP	0.747 (0.720–0.775)	<0.0001	68.9	68.2	2278.3
FLI	0.791 (0.766–0.816)	-	70.9	70.9	29.9358

*p*-values were calculated for ZJU, VAI, HSI, and LAP compared to FLI. MAFLD, metabolic dysfunction-associated fatty liver disease; AUROC, area under the receiver operating characteristic curve; CI, confidence interval; ZJU, Zhejiang University index; VAI, visceral adiposity index; HSI, hepatic steatosis index; LAP, lipid accumulation product; FLI, fatty liver index.

**Table 4:** Sex differences in the diagnostic accuracy of ZJU, VAI, HSI, LAP, and FLI.

	AUROC (95% CI) for Men	*p*-Value	AUROC (95% CI) for Women	*p*-Value
ZJU	0.689 (0.652–0.726)	<0.0001	0.656 (0.597–0.715)	<0.0001
VAI	0.712 (0.675–0.748)	<0.0001	0.671 (0.611–0.731)	<0.0001
HSI	0.773 (0.740–0.806)	<0.0001	0.793 (0.749–0.838)	<0.0001
LAP	0.745 (0.711–0.780)	<0.0001	0.716 (0.664–0.769)	<0.0001
FLI	0.765 (0.732–0.798)	-	0.762 (0.715–0.810)	-

*p*-values were calculated for ZJU, VAI, HSI, and LAP compared to FLI. AUROC, area under the receiver operating characteristic curve; CI, confidence interval; ZJU, Zhejiang University index; VAI, visceral adiposity index; HSI, hepatic steatosis index; LAP, lipid accumulation product; FLI, fatty liver index.

**Table 5:** Diagnostic accuracy of the indices for different age groups.

	AUROC for ≤40 Years (95% CI)	AUROC for 41–50 Years (95% CI)	AUROC for 51–60 Years (95% CI)	AUROC for >60 Years (95% CI)
ZJU	0.759 (0.684–0.834)	0.763 (0.712–0.814)	0.665 (0.617–0.713)	0.651 (0.578–0.724)
VAI	0.765 (0.683–0.847)	0.784 (0.734–0.834)	0.658 (0.609–0.707)	0.628 (0.553–0.704)
HSI	0.862 (0.811–0.913)	0.814 (0.769–0.859)	0.745 (0.702–0.789)	0.748 (0.681–0.816)
LAP	0.783 (0.711–0.856)	0.822 (0.778–0.865)	0.711 (0.666–0.756)	0.667 (0.595–0.739)
FLI	0.838 (0.778–0.899)	0.849 (0.808–0.889)	0.759 (0.717–0.801)	0.713 (0.646–0.781)

AUROC, area under the receiver operating characteristic curve; CI, confidence interval; ZJU, Zhejiang University index; VAI, visceral adiposity index; HSI, hepatic steatosis index; LAP, lipid accumulation product; FLI, fatty liver index.

## Data Availability

The data that support the findings of this study are openly available at: https://knhanes.kdca.go.kr/knhanes/sub03/sub03_01.do (accessed on 30 January 2020).
